# Supplementary material for: A whole-animal phenotypic drug screen identifies suppressors of atherogenic lipoproteins
Source: eLife. 2026 Aug 27;14:RP105314. doi: 10.7554/eLife.105314 (PMC13521566; doi:10.7554/eLife.105314)
Supplement: Supplementary file 2 [file elife-105314-code1.zip › JHDL_primary_result/Fendiline (3,3-Diphenyl-N-1-propanamine)/Fendiline (3,3-Diphenyl-N-1-propanamine)_JHDL_28_030_020_2019_12_06_11_34.pdf]

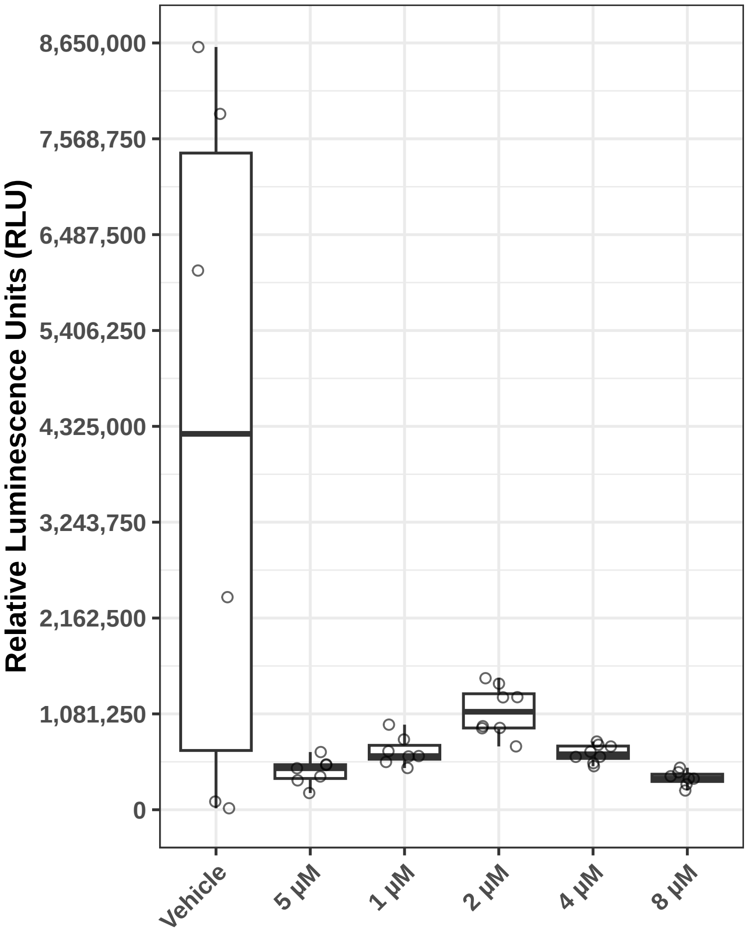
Relative Luminescence Units (RLU)
8,650,000
7,568,750
6,487,500
5,406,250
4,325,000
3,243,750
2,162,500
1,081,250
0
Vehicle
5 µM
1 µM
2 µM
4 µM
8 µM
